# Modified J‐CAPRA scoring system in predicting treatment outcomes of metastatic prostate cancer patients undergoing androgen deprivation therapy

**DOI:** 10.1002/cam4.3548

**Published:** 2020-10-24

**Authors:** Jasmine Lim, Shiro Hinotsu, Mizuki Onozawa, Rohan Malek, Murali Sundram, Guan C. Teh, Teng‐Aik Ong, Shankaran Thevarajah, Rohana Zainal, Say C. Khoo, Shamsuddin Omar, Noor A. Nasuha, Hideyuki Akaza

**Affiliations:** ^1^ Department of Surgery Faculty of Medicine University of Malaya Kuala Lumpur Malaysia; ^2^ Department of Biostatistics and Clinical Epidemiology Sapporo Medical University Hokkaido Japan; ^3^ Department of Urology School of Medicine International University of Health and Welfare Chiba Japan; ^4^ Department of Urology Selayang Hospital Ministry of Health Malaysia Selangor Malaysia; ^5^ Department of Urology Kuala Lumpur Hospital Ministry of Health Malaysia Kuala Lumpur Malaysia; ^6^ Department of Urology Sarawak General Hospital Ministry of Health Malaysia Kuching Malaysia; ^7^ Department of Surgery Queen Elizabeth Hospital Ministry of Health Malaysia Kota Kinabalu Malaysia; ^8^ Department of Surgery Sultanah Bahiyah Hospital Ministry of Health Malaysia Alor Setar Malaysia; ^9^ Department of Urology Penang Hospital Ministry of Health Malaysia Penang Malaysia; ^10^ Department of Urology Sultanah Aminah Hospital Ministry of Health Malaysia Johor Bahru Malaysia; ^11^ Department of Surgery Raja Perempuan Zainab II Hospital Ministry of Health Malaysia Kota Bahru Malaysia; ^12^ Strategic Investigation on Comprehensive Cancer Network Interfaculty Initiative in Information Studies/Graduate School of Interdisciplinary Information University of Tokyo Tokyo Japan

**Keywords:** advanced prostate cancer, overall survival, progression‐free survival, risk stratification, treatment response

## Abstract

The J‐CAPRA score is an assessment tool which stratifies risk and predicts outcome of primary androgen deprivation therapy (ADT) using prostate‐specific antigen, Gleason score, and clinical TNM staging. Here, we aimed to assess the generalisability of this tool in multi‐ethnic Asians. Performance of J‐CAPRA was evaluated in 782 Malaysian and 16,946 Japanese patients undergoing ADT from the Malaysian Study Group of Prostate Cancer (M‐CaP) and Japan Study Group of Prostate Cancer (J‐CaP) databases, respectively. Using the original J‐CAPRA, 69.6% metastatic (M1) cases without T and/or N staging were stratified as intermediate‐risk disease in the M‐CaP database. To address this, we first omitted clinical T and N stage variables, and calculated the score on a 0–8 scale in the modified J‐CAPRA scoring system for M1 patients. Notably, treatment decisions of M1 cases were not directly affected by both T and N staging. The J‐CAPRA score threshold was adjusted for intermediate (modified J‐CAPRA score 3–5) and high‐risk (modified J‐CAPRA score ≥6) groups in M1 patients. Using J‐CaP database, validation analysis showed that overall survival, prostate cancer‐specific survival, and progression‐free survival of modified intermediate and high‐risk groups were comparable to those of original J‐CAPRA (*p* > 0.05) with Cohen's coefficient of 0.65. Around 88% M1 cases from M‐CaP database were reclassified into high‐risk category. Modified J‐CAPRA scoring system is instrumental in risk assessment and treatment outcome prediction for M1 patients without T and/or N staging.

## Graphical abstract

The modified J‐CAPRA scoring system is a risk assessment tool to predict the outcome of primary androgen deprivation therapy (ADT) based on Gleason score, prostate‐specific antigen, and clinical M stage alone. It is beneficial and applicable in stratifying the risk and predicting the treatment outcomes of M1 patients without T and/or N staging.

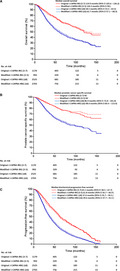

## 1 INTRODUCTION

Prostate cancer incidence varies markedly across the globe, attributing to population genetics, dietary intake, access to healthcare, local screening program, and diagnostic practices. Similar trend was observed in Asia. For instance, the incidence rate of prostate cancer in Japan (age‐standardised rate, ASR 35.4 per 100,000) was higher than Malaysia (ASR 12.4 per 100,000). However, Malaysian had a higher mortality rate (ASR 5.6 per 100,000) compared to Japanese (ASR 4.4 per 100,000),^1^ resulting from ~60% of patients diagnosed at locally advanced and metastatic stages in Malaysia as well as limited access to survival‐prolonging treatments.^2^, ^3^

Androgen‐deprivation therapy (ADT) is the mainstay of first‐line treatment for metastatic prostate cancer.^4^ Combination of radical prostatectomy or radiotherapy with ADT improved the survival of patients with high‐risk localised or locally advanced disease.^5^, ^6^ Of note, high‐risk localised prostate cancer includes patients with prostate‐specific antigen (PSA) >20 ng/ml or Gleason score >7 (Gleason Grade Group 4/5) or cT2c, while those with cT3‐4 or cN+ (any PSA and Gleason score) diseases are classified as locally advanced.^7^ High proportion of advanced disease entails special challenge in predicting the disease risk of patients receiving ADT.

The J‐CAPRA (Japan Cancer of the Prostate Risk Assessment) score is a novel, validated risk assessment tool in predicting outcomes of ADT in prostate cancer patients. This multivariable risk assessment tool was constructed from Japan Study Group Prostate Cancer (J‐CaP) 2001–2003 database, which is a national registry of prostate cancer patients undergoing ADT in Japan.^8^ It is applicable to the entire spectrum of risk and stage, both localised and advanced prostate cancer.^8^ Therefore, the objective of this study was to assess the generalisability of J‐CAPRA scoring system in stratifying the risk of prostate cancer patients from a multi‐ethnic Asian population.

## 2 MATERIALS AND METHODS

The M‐CaP comprised patients accrued from nine urology referral centers across Malaysia, with 15.2% of patients treated at academic center. A total of 1152 men were recruited into the cohort between 2016 and May 2018, of whom 578 underwent primary ADT and 204 received ADT combined with radiation, surgery or chemotherapy. Type of ADT was categorised into orchidectomy, luteinizing hormone‐releasing hormone (LHRH) agonist monotherapy, or LHRH agonist with anti‐androgen (bicalutamide 50 mg daily) [maximum androgen blockade (MAB)]. Men receiving anti‐androgen monotherapy were excluded from this study. Data on initial and subsequent PSA levels, TNM staging, treatment patterns, disease progression, and mortality were documented prospectively with a written form (proforma) based on medical, radiological, and pathological records. Details from the proforma were then transformed into an electronic database for further analysis. Patients were followed under specialist review every 3 months.

The J‐CaP 2001–2003 database contained 26,272 prostate cancer patients diagnosed during 2001–2003 and received ADT, either as primary treatment or in combination with radiotherapy or surgery across 384 institutions in Japan.^9^, ^10^ The database represented 95% of Japanese patients treated with primary ADT in the academic centers or community hospitals. Clinical stage, treatment patterns, disease progression, all‐cause, and prostate cancer‐specific mortality (CSM) were ascertained from participating urologists on a quarterly basis. In this study, 16,946 patients treated with primary ADT were included for analysis. Men receiving anti‐androgen as monotherapy were excluded. Ethical approval for the M‐CaP and J‐CaP databases was granted by the Medical Research and Ethics Committee (MREC), Ministry of Health Malaysia, and regional Medical Research Review Board, respectively.

### 2.1 Statistical analysis

Clinical and demographic characteristics were compared between M‐CaP and J‐CaP 2001–2003 databases with Student's *t*‐test and chi‐square test, as appropriate. Of note, the J‐CaP 2001–2003 was used in the initial development of J‐CAPRA score.^8^ The disease risk of each man in M‐CaP was assessed based on the J‐CAPRA. The validated J‐CAPRA scoring system uses a 12‐point scale based on five parameters including PSA level (up to 3 scores), biopsy Gleason score (up to 2 scores), clinical T stage (up to 3 scores), clinical N stage (up to 1 score), and clinical M stage (up to 3 scores).^8^ Validated score groups were introduced to stratify low‐risk (J‐CAPRA score 0–2), intermediate‐risk (J‐CAPRA score 3–7), and high‐risk (J‐CAPRA score ≥8) groups.^8^

Performance of J‐CAPRA was evaluated across all disease stages using M‐CaP and J‐CaP 2001–2003 databases. First, we examined the risk scores distribution of M‐CaP and J‐CaP 2001–2003 databases. Second, we assessed the differences in risk scores distribution between J‐CaP and M‐CaP across Union for International Cancer Control (UICC) stage I‐IV groups. The J‐CAPRA was further modified by omitting T and N stage score points for the M1 group. To validate the J‐CAPRA modifications for the M1 group, overall survival, prostate cancer‐specific survival, and progression‐free survival of J‐CaP 2001–2003 were compared between risk categories defined by original J‐CAPRA and modified J‐CAPRA, through Kaplan–Meier survival curves and Cohen's coefficient of agreement. Overall survival, prostate cancer‐specific survival, and progression‐free survival were defined as time from initiation of ADT to death, to death from disease, and to the first event of radiological and/or biochemical progression or death, respectively. Statistical analysis was performed using SPSS for Windows version 21.0 (SPSS Inc.). Two‐tailed *p* value <0.05 was termed as statistically significant.

## 3 RESULTS

There were 16,946 patients in J‐CaP and 782 patients in M‐CaP receiving ADT, either as primary ADT or in combination with radiotherapy, surgery or chemotherapy. Summary of clinical characteristics of both J‐CaP and M‐CaP cohorts is presented in Table 1. Patients receiving ADT in J‐CaP were older than those in M‐CaP with an average age of 75 ± 7.2 years versus 69.6 ± 7.7 years (*p* < 0.01, Student's *t*‐test). Patients in M‐CaP had a higher burden of comorbidity compared to the J‐CaP cohort (*p* < 0.01, chi‐square test). Maximum androgen blockade (67%) was the most common treatment, followed by orchidectomy (23.2%) and LHRH agonist monotherapy (9.8%) in the J‐CaP cohort. The M‐CaP cohort had relatively higher‐risk disease compared to J‐CaP cohort, with a median PSA level of 99 ng/ml (interquartile range, IQR 31.1–352.5 ng/ml) versus 27.2 ng/ml (IQR 10.5–109.2 ng/ml), a median biopsy Gleason score of 8 versus 7 and a higher percentage of UICC stage IV cases at 74.1% versus 35.4%.

**TABLE 1:** Baseline patient characteristics

Factors	Frequency distribution, *n* (%)		*p* value
	J‐CaP (*n* = 16,946)	M‐CaP (*n* = 782)	
Age (y)	75.0 ± 7.2	69.6 ± 7.7	<0.01
PSA at diagnosis (ng/ml)			
0–20	7258 (43.0)	119 (15.4)	<0.01
>20−100	5264 (31.1)	270 (34.9)	
>100−500	2614 (15.5)	233 (30.1)	
>500	1759 (10.4)	151 (19.6)	
*unknown*	51	9	
Biopsy Gleason score			
≤6	5027 (34.1)	53 (7.2)	<0.01
7	4281 (29.1)	224 (30.2)	
8–10	5414 (36.8)	464 (62.6)	
*unknown*	2224	41	
UICC Staging system			
Stage I	5452 (33.5)	20 (2.6)	<0.01
Stage II	2056 (12.6)	79 (10.2)	
Stage III	3070 (18.8)	101 (13.1)	
Stage IV	5723 (35.1)	572 (74.1)	
*unknown*	645	10	
Comorbidity count			
0	5797 (34.2)	205 (26.2)	<0.01
1	5700 (33.6)	173 (22.1)	
2	3417 (20.2)	203 (26.0)	
3	1445 (8.5)	140 (17.9)	
≥4	587 (3.5)	61 (7.8)	
ADT type			
Orchidectomy	3935 (23.2)	105 (13.5)	<0.01
LHRH agonist	1656 (9.8)	616 (79.3)	
MAB	11355 (67.0)	56 (7.2)	
*unknown*	0	5	

Abbreviations: ADT, androgen deprivation therapy; LHRH, luteinizing hormone‐releasing hormone; MAB, maximum androgen blockade; PSA, prostate‐specific antigen; UICC, Union for International Cancer Control.

**FIGURE 1:**
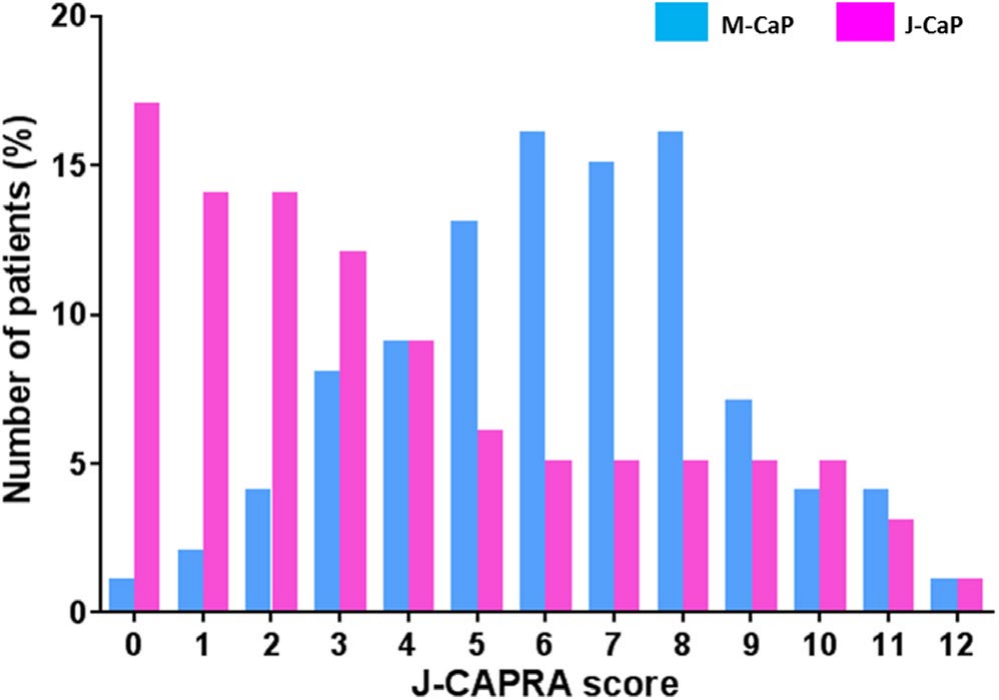
Distribution of J‐CAPRA score in the M‐CaP and J‐CaP databases

For J‐CAPRA scoring, a total of 734 men in M‐CaP with complete risk stratification data including PSA level, biopsy Gleason Score, and disease staging were recruited into the analysis. The J‐CAPRA score distribution of M‐CaP is summarised in Figure 1. Most (60.9%) had a J‐CAPRA score of 3–7, while 31.7% had a score greater than 8 and 7.4% had a score less than 2. Conversely, 81.2% were with J‐CAPRA score ≤7 and 18.8% had a J‐CAPRA score ≥8 in the J‐CaP database (Figure 1). Risk stratification of J‐CAPRA scores showed that 36/97 (37.1%) Stage I and II cases were low‐risk disease (J‐CAPRA score 0–2), while 87/101 (86.1%) Stage III cases were classified as intermediate‐risk (J‐CAPRA score 3–7) (Table 2A). Interestingly, only 43.5% (233/536) of Stage IV cases were of high risk (J‐CAPRA score ≥8) (Table 2A). Comparing to the J‐CAP 2001–2003 database, a relatively higher proportion (57%; 2604/4572) of Stage IV cases were stratified into the high‐risk group (J‐CAPRA score ≥8) (Table 2B). Majority (82.8%; 5427/6552) of the Stage I and II cases were classified as low risk, while 75.4% (2071/2754) Stage III cases were intermediate‐risk disease in the J‐CaP 2001–2003 database.

**TABLE 2:** Patient distribution of all disease stages across low, intermediate, and high‐risk groups based on original J‐CAPRA scores in the M‐CaP (A) and J‐CaP (B) databases

Original J‐CAPRA risk group	UICC Staging				Total, *n* (%)
	I	II	III	IV	
(A) M‐CaP					
Low (0−2)	16	20	14	4	54 (7.4)
Intermediate (3−7)	3	58	87	299	447 (60.9)
High (≥8)	0	0	0	233	233 (31.7)
Total	19	78	101	536	734 (100)
(B) J‐CaP					
Low (0−2)	4439	988	674	34	6135 (44.2)
Intermediate (3−7)	310	815	2071	1934	5130 (37.0)
High (≥8)	0	0	0	2604	2604 (18.8)
Total	4749	1803	2745	4572	13,869 (100)

Further analysis revealed that 208/299 (69.6%) of stage IV metastatic (M1) cases without T and/or N staging were grouped into intermediate‐risk (J‐CAPRA score 3–7) category in the M‐CaP database. Of note, these M1 patients did not undergo T and/or N staging as it did not affect treatment decisions directly.^7^ All M1 patients in the J‐CaP 2001–2003 database had complete clinical T, N, and M staging.

**FIGURE 2:**
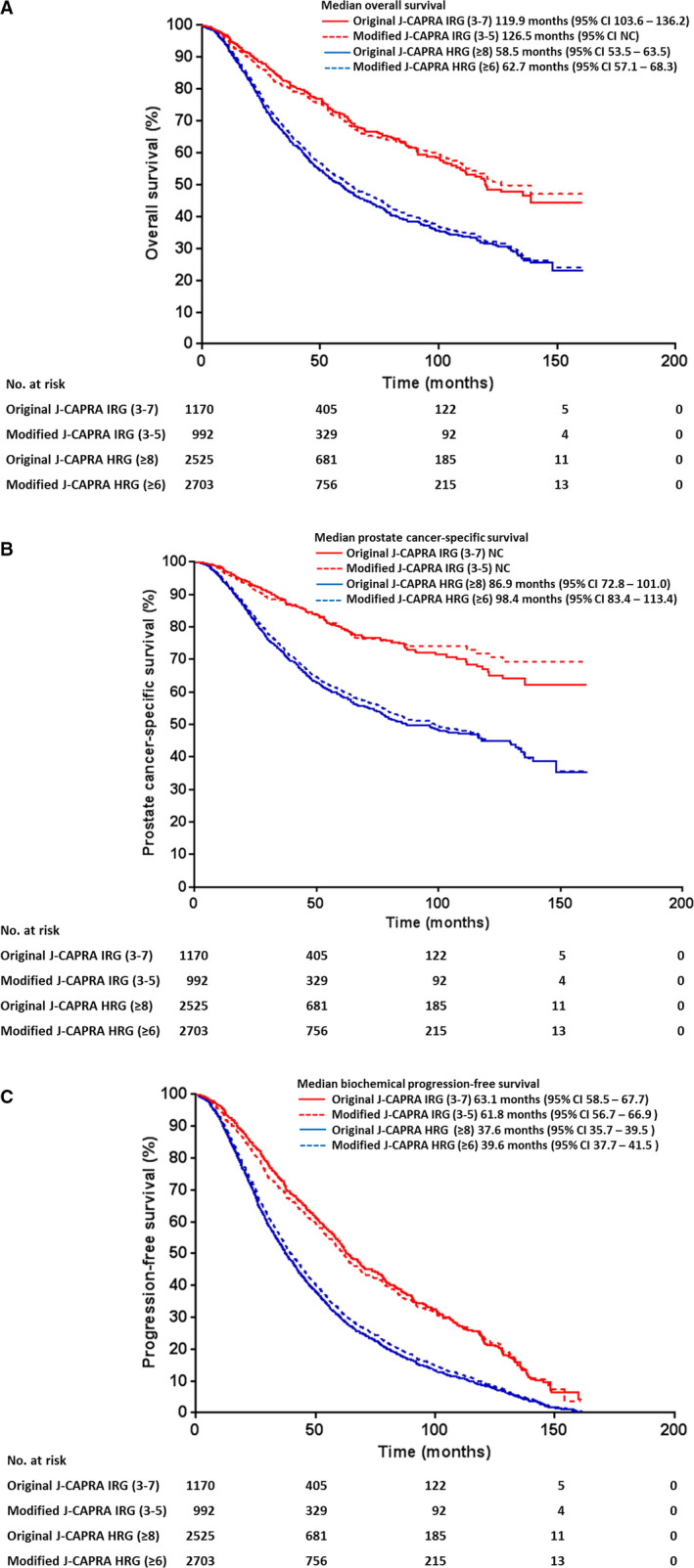
The (A) overall survival, (B) prostate cancer‐specific survival, and (C) progression‐free survival for original J‐CAPRA intermediate‐risk group, IRG (3–7) and high‐risk group, HRG (≥8) and modified J‐CAPRA intermediate (3–5) and high‐risk (≥6) groups among metastatic (M1) prostate cancer patients

To address this, we first omitted clinical T and N stage variables, and calculated the score on a 0–8 rather than 0–12 scale in the modified J‐CAPRA scoring system for M1 patients. The J‐CAPRA score threshold was further adjusted for intermediate (modified J‐CAPRA score 3–5) and high‐risk (modified J‐CAPRA score ≥6) groups for M1 patients, based on biopsy Gleason score, PSA, and clinical M stage alone. Using the J‐CaP 2001–2003 database, we performed sensitivity analyses to measure whether there were discrepancies of original J‐CAPRA intermediate (3–7) and high‐risk (≥8) groups against modified J‐CAPRA intermediate (3–5) and high‐risk (≥6) groups in the overall survival, prostate cancer‐specific survival, and progression‐free survival. We demonstrated that there were no significant difference in the median overall survival and prostate cancer‐specific survival of M1 patients between original J‐CAPRA intermediate and high‐risk group and modified J‐CAPRA intermediate and high‐risk group (*p* > 0.05, Log‐rank test) (Figure 2A,B). A comparable median progression‐free survival (PFS) of M1 patients was observed in both intermediate (original J‐CAPRA score 3–7; PFS 63.1 months, 95% CI 58.5–67.7 versus modified J‐CAPRA 3–5; PFS 61.8 months, 95% CI 56.7–66.9) and high‐risk groups (original J‐CAPRA score ≥8; PFS 37.6 months, 95% CI 35.7–39.5 versus modified J‐CAPRA score ≥6; PFS 39.6 months, 95% CI 37.7–41.5) stratified by original and modified J‐CAPRA scoring systems (*p* > 0.05, Log‐rank test) (Figure 2C). The agreement of J‐CAPRA score and modified J‐CAPRA score among M1 patients was further supported by Cohen's coefficient of 0.65. Using the modified J‐CAPRA scores, 227/299 (75.9%) of Stage IV cases in the M‐CaP database were re‐grouped from intermediate‐risk category into high‐risk category (Table 3); of which, 87.7% (199/227) were M1 patients.

**TABLE 3:** Patient distribution of all disease stages across low, intermediate, and high‐risk groups based on modified J‐CAPRA scores in the M‐CaP database

Modified J‐CAPRA risk group	UICC Staging				Total, *n* (%)
	I	II	III	IV	
Low (0−2)	16	20	14	4	54 (7.4)
Intermediate (3−5)	3	58	87	72	220 (30.0)
High (≥6)	0	0	0	460	460 (62.7)
Total	19	78	101	536	734 (100)

## 4 DISCUSSION

Findings from this study demonstrated that prostate cancer patients receiving ADT in Malaysia tend to be younger with higher risk and more advanced tumors than those treated in Japan. In the present study, we report for the first time the generalizability of the J‐CAPRA scoring system in a multi‐ethnic Asian cohort. We made two modifications to the J‐CAPRA scoring system in assessing the risk of metastatic prostate cancer patients undergoing ADT by omitting clinical T and N staging.

Risk‐stratification for prostate cancer is pivotal to guide appropriate treatment decision making at diagnosis and subsequent decision points. Using the Cancer of the Prostate Strategic Urologic Research Endeavor (CaPSURE) database in United States,^11^ the Cancer of the Prostate Risk Assessment (CAPRA) scoring system was established to incorporate simplicity and clinical applicability in the nomogram performance for assessing risk of radical prostatectomy patients.^12^ Multiple independent studies have been conducted for validating the CAPRA score.^13^

With high burden of advanced disease, risk stratification of the M‐CaP population meets unusual challenges. Standard risk stratification schemata apply for localised disease only.^14^, ^15^ The J‐CAPRA score estimates risk of progression‐free survival and prostate cancer‐specific mortality for locally advanced and metastatic prostate cancer patients undergoing ADT. Validation of J‐CAPRA score has been performed in numerous academic institutions worldwide.^13^ Based on the J‐CAPRA score risk stratification, it also showed that men on ADT in J‐CaP had a lower prostate cancer‐specific mortality rate compared to those in the USA, although Japanese had higher disease risk profiles.^16^

The modified J‐CAPRA scoring system is a good and simple model requiring PSA level, biopsy Gleason score, and clinical M staging alone, which are easily assessable from a daily consultation setting, for metastatic prostate cancer patients undergoing ADT. Based on the validation analysis in a large J‐CaP cohort (>10,000 subjects), it appears to perform well in predicting disease progression in patients of different risk groups and achieve substantial Cohen's coefficient agreement of 0.65 with J‐CAPRA scoring system. A relatively much lower M1 case (15.2%) in the J‐CaP database were re‐grouped from J‐CAPRA intermediate‐risk group to modified J‐CAPRA high‐risk group compared to M1 cases (88%) in the M‐CaP database as all J‐CAPRA‐scored M1 cases of J‐CaP database had complete TNM staging. This finding also suggests the comparability of modified J‐CAPRA to J‐CAPRA scoring system without the presence of T and N staging. Modified J‐CAPRA scoring system is applicable for risk stratification of metastatic prostate cancer patients from other low‐ and middle‐income countries with limited healthcare resources. While cost is a major issue for prostate cancer patients from these countries, complete TNM staging for M1 cases is unlikely to be performed in these countries since both T and N staging do not affect treatment decisions directly in M1 cases. High‐risk prostate cancer patients receiving primary ADT may develop rapid disease progression and benefit from early enrolment into clinical trials of novel androgen‐receptor targeted drugs, chemotherapy, radiopharmaceutical agent, immunotherapy or combination therapies.

There are limitations to this analysis. First, we were unable to address the 5‐years median overall survival, prostate cancer‐specific survival, and progression‐free survival of M‐CaP database as its median follow‐up time is currently less than 5 years; therefore, a comparison of outcomes between the J‐CaP and M‐CaP cohorts could not be established at this time. Second, it remains unknown whether these findings can be extrapolated to other populations. A multi‐institutional analysis involving other developing nations is currently underway. Third, there could be a random error in the reporting of disease progression in the J‐CaP study by clinicians owing to various definitions of progression.^8^ Fourth, addition of CHAARTED^17^, ^18^, ^19^ and LATITUDE^20^ criteria such as visceral metastasis and number of bone lesions may increase the accuracy of modified J‐CAPRA scoring system in classifying the risk of prostate cancer. Nevertheless, our intention was to establish a risk assessment tool that is simple and quick to be utilised in a routine clinic consultation setting particularly in low‐ and middle‐income countries with limited healthcare resources.

In summary, the J‐CAPRA scoring system is developed to aid clinicians and patients in predicting ADT treatment outcomes of prostate cancer. We have modified and validated the J‐CAPRA scoring system in order to stratify the risk of M1 patients without T and/or N staging. It is essential to confirm and validate these findings in other developing nations as healthcare cost remains a key element in deciding the management of prostate cancer.

## CONFLICT OF INTEREST

All authors declare no conflict of interest.

## Data Availability

The data used to support the findings of this study are available from the corresponding author upon request.

## References

[1] GLOBOCAN 2018: Estimated cancer incidence, mortality and prevalence worldwide in 2018. http://gcoiarcfr

[2] Summary of Malaysian National Cancer Registry Report 2007–2011. http://ncimohgovmy

[3] Saad M , Alip A , Lim J , et al. Management of advanced prostate cancer in a middle‐income country: real‐world consideration of the Advanced Prostate Cancer Consensus Conference 2017. BJU Int. 2019;124:373‐382. 31077523 10.1111/bju.14807 PMC6851975

[4] Huggins C , Hodges CV . Studies on prostatic cancer. I. The effect of castration, of estrogen and androgen injection on serum phosphatases in metastatic carcinoma of the prostate. Cancer Res. 1941;1:293‐297. 10.3322/canjclin.22.4.232 4625049

[5] Mason MD , Parulekar WR , Sydes MR , et al. Final report of the intergroup randomized study of combined androgen‐deprivation therapy plus radiotherapy versus androgen‐deprivation therapy alone in locally advanced prostate cancer. J Clin Oncol. 2015;33:2143‐2150. 25691677 10.1200/JCO.2014.57.7510 PMC4477786

[6] Warde P , Mason M , Ding K , et al. Combined androgen deprivation therapy and radiation therapy for locally advanced prostate cancer: a randomised, phase 3 trial. Lancet. 2011;378:2104‐2111. 22056152 10.1016/S0140-6736(11)61095-7 PMC3243932

[7] EAU Guidelines: Edn. presented at the EAU Annual Congress Amsterdam, 2020. ISBN 978‐94‐92671‐07‐3.

[8] Cooperberg MR , Hinotsu S , Namiki M , et al. Risk assessment among prostate cancer patients receiving primary androgen deprivation therapy. J Clin Oncol. 2009;27:4306‐4313. 19667269 10.1200/JCO.2008.21.5228 PMC2744272

[9] Akaza H , Usami M , Hinotsu S , et al. Characteristics of patients with prostate cancer who have initially been treated by hormone therapy in Japan: J‐CaP surveillance. Jpn J Clin Oncol. 2004;34:329‐336. 15333685 10.1093/jjco/hyh061

[10] Hinotsu S , Akaza H , Usami M , et al. Current status of endocrine therapy for prostate cancer in Japan analysis of primary androgen deprivation therapy on the basis of data collected by J‐CaP. Jpn J Clin Oncol. 2007;37:775‐781. 17965423 10.1093/jjco/hym098

[11] Lubeck DP , Litwin MS , Henning JM , et al. The CaPSURE database: a methodology for clinical practice and research in prostate cancer. CaPSURE Research Panel. Cancer of the Prostate Strategic Urologic Research Endeavor. Urology. 1996;48:773‐777. 8911524 10.1016/s0090-4295(96)00226-9

[12] Cooperberg MR , Pasta DJ , Elkin EP , et al. The University of California, San Francisco Cancer of the Prostate Risk Assessment score: a straightforward and reliable preoperative predictor of disease recurrence after radical prostatectomy. J Urol. 2005;173:1938‐1942. 15879786 10.1097/01.ju.0000158155.33890.e7 PMC2948569

[13] Brajtbord JS , Leapman MS , Cooperberg MR . The CAPRA Score at 10 years: contemporary perspectives and analysis of supporting studies. Eur Urol. 2017;71:705‐709. 27616723 10.1016/j.eururo.2016.08.065

[14] Thurtle D , Rossi SH , Berry B , Pharoah P , Gnanapragasam VJ . Models predicting survival to guide treatment decision‐making in newly diagnosed primary non‐metastatic prostate cancer: a systematic review. BMJ Open. 2019;9:e029149. 10.1136/bmjopen-2019-029149 PMC6596988 31230029

[15] Shariat SF , Karakiewicz PI , Roehrborn CG , Kattan MW . An updated catalog of prostate cancer predictive tools. Cancer. 2008;113:3075‐3099. 18823041 10.1002/cncr.23908

[16] Cooperberg MR , Hinotsu S , Namiki M , Carroll PR , Akaza H . Trans‐Pacific variation in outcomes for men treated with primary androgen‐deprivation therapy (ADT) for prostate cancer. BJU Int. 2016;117:102‐109. 25238114 10.1111/bju.12937

[17] Sweeney CJ , Chen Y‐H , Carducci M , et al. Chemohormonal therapy in metastatic hormone‐sensitive prostate cancer. N Engl J Med. 2015;373:737‐746. 26244877 10.1056/NEJMoa1503747 PMC4562797

[18] Kyriakopoulos CE , Chen Y‐H , Carducci MA , et al. Chemohormonal therapy in metastatic hormone‐sensitive prostate cancer: long‐term survival analysis of the randomized phase III E3805 CHAARTED trial. J Clin Oncol. 2018;36:1080‐1087. 29384722 10.1200/JCO.2017.75.3657 PMC5891129

[19] Gravis G , Boher J‐M , Chen Y‐H , et al. Burden of metastatic castrate naive prostate cancer patients, to identify men more likely to benefit from early docetaxel: further analyses of CHAARTED and GETUG‐AFU15 studies. Eur Urol. 2018;73:847‐855. 29475737 10.1016/j.eururo.2018.02.001 PMC6010352

[20] Fizazi K , Tran NP , Fein L , et al. Abiraterone plus prednisone in metastatic, castration‐sensitive prostate cancer. N Engl J Med. 2017;377:352‐360. 28578607 10.1056/NEJMoa1704174

